# Novel Catalytic Ceramic Conversion Treatment of Ti6Al4V for Improved Tribological and Antibacterial Properties for Biomedical Applications

**DOI:** 10.3390/ma14216554

**Published:** 2021-11-01

**Authors:** James Alexander, Huan Dong, Deepa Bose, Ali Abdelhafeez Hassan, Sein Leung Soo, Zhenxue Zhang, Xiao Tao, Sarah Kuehne, Xiaoying Li, Hanshan Dong

**Affiliations:** 1 School of Metallurgy and Materials, University of Birmingham, Birmingham B15 2TT, UK; JXA387@student.bham.ac.uk (J.A.); z.zhang.1@bham.ac.uk (Z.Z.); X.Tao@bham.ac.uk (X.T.); h.dong.20@bham.ac.uk (H.D.); 2 Queen Elizabeth Hospital, Birmingham B15 2WB, UK; huan.dong@nhs.net (H.D.); deepabose@yahoo.com (D.B.); 3 School of Engineering, University of Birmingham, Birmingham B15 2TT, UK; A.M.AbdelhafeezHassan@bham.ac.uk (A.A.H.); s.l.soo@bham.ac.uk (S.L.S.); 4 School of Dentistry, University of Birmingham, Birmingham B5 7EG, UK; S.A.Kuehne@bham.ac.uk

**Keywords:** ceramic conversion, Titanium oxide, surface hardening of Ti alloys, biomechanical properties, Ti fixation pins, antibacterial behaviour

## Abstract

Titanium oxide layers were produced via a novel catalytic ceramic conversion treatment (CCCT, C3T) on Ti-6Al-4V. This CCCT process is carried out by applying thin catalytic films of silver and palladium onto the substrate before an already established traditional ceramic conversion treatment (CCT, C2T) is carried out. The layers were characterised using scanning electron microscopy, X-ray diffraction, transmission electron microscopy; surface micro-hardness and reciprocating tribological performance was assessed; antibacterial performance was also assessed with *S. aureus*. This CCCT has been shown to increase the oxide thickness from ~5 to ~100 µm, with the production of an aluminium rich layer and agglomerates of silver and palladium oxide surrounded by vanadium oxide at the surface. The wear factor was significantly reduced from ~393 to ~5 m^3^/N·m, and a significant reduction in the number of colony-forming units per ml of *Staphylococcus aureus* on the CCCT surfaces was observed. The potential of the novel C3T treatment has been demonstrated by comparing the performance of C3T treated and untreated Ti6Al4V fixation pins through inserting into simulated bone materials.

## 1. Introduction

Due to their high strength-to-weight ratio and superior corrosion resistance, titanium alloys have been adopted for a wide range of applications. Especially within the aerospace industry for which Ti-6Al-4V was the first developed workhorse. Since then, it has been shown to have attractive and beneficial effects within the biomedical field in terms of satisfactory biocompatibility and desirable osseointegration for orthopaedic and dental implants. In addition to this, its low elastic modulus aids in reducing the stress-shielding phenomenon and therefore could reduce the number of failures due to aseptic loosening of load-bearing prosthesis [1,2,3].

However, titanium (Ti) and its alloys have poor tribological properties as they are prone to galling. This is mainly because of their low hardness, ease of plastic deformation, and high reactivity, thus causing strong adhesion and even seizure. To make matters worse, the poor wear properties of Ti leads to the formation of Ti wear debris from contacting surfaces. For example, galvanic corrosion and fretting wear occurred at the Ti stem/CoCr head taper connection of modular designed hip prosthesis when the naturally formed very thin TiO_2_ film was removed by fretting [4]. Wear debris can also be produced during the insertion of the fixture of dental implants [5] and self-drilling fixation pins for external fracture fixation [6].

Metal debris is the main reason for the release of titanium ions due to the dissolution of wear particles. This may lead to tissue discolouration and/or elicit an adverse inflammatory response, which will cause inflammation, peri-implantitis and eventually aseptic implant loosening [7,8]. In addition to these local implications, the release of ions into the surrounding tissue can result in systemic implications. This is because if the particles/ions diffuse through the tissue, they can get distributed through the lymphatic system, and therefore accumulate in the liver and spleen [9,10]. Thus, how to improve the tribological properties of Ti implants and reduce wear debris induced metal ion release presents a major challenge to the performance and longevity of Ti implants. To this end, great efforts have been made to coat Ti implants with bio-inert hard ceramic coatings (such as CrN & DLC) or bio-active coatings (such as HA). However, spallation of coatings due to poor interfacial bonding and low load supporting of the soft Ti substrate has cast a shadow on the successful use of coatings for load supporting Ti implants.

A recently developed ceramic conversion treatment (C2T) can address the concerns over thin coatings by in-situ converting a Ti surface into a wear resistant and bio-active TiO_2_ ceramic layer via thermal oxidation, which is supported by an oxygen diffusion hardened subsurface [11] and has shown some promise for improving the wear resistance of Ti fixation pins [12]. The C2T normally takes about 80–100 h at 600–620 °C to produce a surface oxide layer approximately 2–3 microns with good bonding to the substrate. This not only leads to high energy consumption and low production efficiency, but also causes the loss of bulk material strength due to over aging. In addition, it is impossible to produce a thick (>4 microns) TiO_2_ layer without causing spallation of the oxide layer due to poor bonding.

Another major challenge for premature implant failure is due to implant-associated infection [13]. Although there is not much literature published on the true cost of these infections, they result in the need for revision surgery, which not only reduces the patient’s quality of life but also puts a major financial strain on the NHS. Both surface topography and chemistry influence the response of host cell and bacterial adhesion to implant surfaces. One of the methods to reduce the amount of bacterial adhesion is to alter the surface chemistry by applying antimicrobial substances to said surface. A well-documented antimicrobial substance is silver, which in the presence of water/body fluids releases silver cations (Ag+). This Ag+ has an affinity for sulphydryl groups and proteins on cell membranes. Ag+ is then able to alter bacterial cell membranes thus influencing permeability and causing intracellular uptake of Ag+. Then once inside the cells, the Ag+ can interact with subcellular components and therefore impair cellular metabolism and replication [13,14,15]. However, the major concern over Ag-containing coatings is failure of the coating, fast leaching of Ag ions, and the resulting toxicity [16]. Although C2T surfaces demonstrate a certain level of antimicrobial capability, it should be noted that the efficacy is most probably too low to effectively combat post-implantation infection.

To this end, this paper reports, for the first time, a novel surface engineering technology to generate a thick TiO_2_ oxide layer with both high wear resistance and antimicrobial efficacy within a short period of time based on catalytic ceramic conversion treatment (C3T) by pre-depositing a thin catalysing layer consisting of Ag and Pd prior to ceramic conversion treatment. The surface cases formed on Ti6Al4V by C3T were fully characterised, the wear and antibacterial efficacy evaluated, and the potential of the novel C3T treatment has been demonstrated by comparing the performance of C3T treated and untreated Ti6Al4V fixation pins through insertion into simulated bone materials. Based on the results, possible mechanisms of the catalytic effect of Ag/Pd on the fast growth of a good quality surface oxide layer is discussed. This reported new study, from a scientific point-of-view, advanced scientific understanding of the mechanism involved and, from technological point of view, paves the way for biomedical applications.

## 2. Materials and Methods

### 2.1. Coupons and Fixation Pins

Ti-6Al-4V coupon samples with a thickness of 4 mm were prepared from a hot-rolled and annealed bar (supplied by IMI Titanium Ltd., Birmingham, UK) of one inch in diameter. The coupon samples were ceramic conversion treated and used for microstructural and property characterisation. Optimised conditions derived from the characterisation of treated coupon samples was selected to treat the commercial Ti6Al4V titanium alloy self-drilling/self-tapping pins (Apex 5018-6-150S, Stryker, MI, USA). The diameter of the pin, total length and thread length were 5, 150 and 50 mm, respectively.

### 2.2. Coatings of Catalytic Layer and Ceramic Conversion Treatments

A catalytic layer of Ag, Pd and Ag + Pd, was coated on Ti64 coupons via a Closed Field Unbalanced Magnetron Sputter Ion Plating (CFUMSIP) system (Teer Coatings, Droitwich, UK). The coatings were carried out in an argon environment with silver, and/or palladium targets, at a current of 1 A and a bias voltage of 40 V. Pulsed mode was utilised at a frequency of 50 kHz and pulse width of 1500 ns. To enable a stable plasma the current was ramped up from 0.3 to 1 A in 30 s. The coating processing time was 4 min, which yields a deposition coating layer of ≈330 nm on the surfaces of the coupons and the pins. The respective coverage of the deposited Ag, Pd and Ag/Pd on the surfaces of the coupons were observed after the coating processes and it was revealed that the deposited layers were homogeneously distributed on the surfaces.

Based on a patented [17] treatment, Ceramic Conversion Treatment, CCT or C2T was carried out on coupon samples with and without catalytic coatings. This converts the titanium surface into a ceramic oxide layer and introduces oxygen into the subsurface to form an oxygen solid solution hardened diffusion case. While the pre-coating layer acts as a catalyst, accelerating the surface ceramic conversion process, named as Catalytic Ceramic Conversion Treatment, CCCT or C3T.

A series of ceramic conversion treatments were carried out to investigate the optimal conditions for producing a surface layer with improved wear resistance and antimicrobial properties. Firstly, samples with and without an Ag/Pd deposition layer were treated at temperatures of 580, 620 and 660 °C for 80 h. After characterisation of the 80 h treated samples in terms of the oxide layer quality, thickness and adhesion to the substrate, 660 °C was selected for a series of treatment durations ranging from 1 to 80 h. Characterisation on these samples revealed a good quality layer for the 5 h treated samples in terms of surface hardness, thickness and interfacial bonding. Further investigations were designed to have individual catalyst coatings of Ag or Pd pre-deposited on the coupons for ceramic conversion treatment of 660 °C/5 h. A controlled atmosphere containing 20% oxygen and 80% nitrogen, at a pressure under 100kPa were constants for all the treatments. Table 1 details the sample codes, denoted in terms of the pre-coating, treatment temperature, and duration etc. Based on the microstructure characterisation and mechanical property evaluation of the coupons, the C3T660-5 with a catalytic layer of Ag + Pd deposition was selected to treat the fixation pins.

### 2.3. Microstructure Characterisation of Treated Coupons and Pins

Treated coupon samples were cut and hot mounted with conductive bakelite. The samples were ground to 1200 grit size with SiC paper and then polished using 6 and then 3 μm diamond suspension, and finally activated colloidal silica suspension. The metallographic microstructures of the samples were revealed using Kroll’s reagent. Surface morphology, layer structure and composition of the coupons were characterised using a JEOL 7000F SEM (Tokyo, Japan) equipped with Energy-dispersive X-ray spectrometer (EDX; Oxford Instruments Inca, Oxford, UK). The surface layer thickness was measured using Image J on SEM images and an average of at least 5 times measurements was taken as the layer thickness. The tip shapes and surface morphology of the pins were optically observed.

The phase composition was analysed using an X-ray diffraction instrument (XRD, D8 Advanced, Bruker, MA, USA), with a Cu source (Kα = 0.154 nm). Further detailed surface layer microstructure and phase composition were analysed for the C3T660-20PdAg sample by transmission electron microscope (TEM). The TEM specimens were prepared perpendicular to the surface by using focused ion beam (FIB, Quanta 3D, FEI company, Oregon, USA) following the standard steps of Pt deposition, bulk-out, u-cut, lift out, mounting, thinning, and cleaning. The microstructure was examined using an electron microscope (LaB6, 200 kV) (2100, Jeol, Tokyo, Japan). High angle annular dark field (HAADF) imaging and Energy-dispersive X-ray spectroscopy (Oxford ISIS EDX, Oxford, UK) analysis were performed using scanning TEM (STEM) mode with a Philips Tecnai F20 instrument (FEG, 200 kV) (Philips Electron Optics, OR, USA).

### 2.4. Mechanical Property Evaluation

Microhardness of the coupons were measured using a MKV-H1 hardness tester (Mitutoyo, Kanagawa, Japan) with a Vickers indenter under the load of 25 and 50 g and the hardness value is an average of five measurements with the standard deviation as the error bars. The tribological performance was measured using a TE79 tribometer (Phoenix Tribology Ltd, Berkshire, UK) with an 8 mm dimeter tungsten carbide counterpart. The reciprocating distance was 5 mm for 1000 cycles, equating to a total 5 m sliding distance. A lighter, 15 N, and heavy, 50 N, load was used to evaluate the tribological properties of untreated and ceramic conversion treated Ti-6Al-4V coupons. The cross-sectional areas of the wear tracks were measured using an Ambios XP-Plus 200 Stylus Profilometer (Ambios Technology, CA, USA), and subsequently, wear volume was calculated by multiplying the cross-sectional area by the length of the wear track. The morphology of the wear tracks was SEM observed.

A comparison of biomechanical properties of Unt and C3T660-5PdAg treated Ti6Al4V pins were evaluated by insertion tests using a cortical bone-simulating material SAWBONES^®^ (Europe AB, Malmö, Sweden) that was delivered as blocks measuring 30 mm × 17 mm × 10 mm. These blocks were used to simulate mechanical pin insertion in actual bone. FX5 machining centre (an industrial drilling machine, Matsuura Machinery Ltd, Coalville, UK) was employed to conduct the insertion tests under a constant feed rate of 70 mm/min and rotational speed of 360 rpm for an insertion depth of 25 mm without pre-drilling through the bone block. The applied axial force needed to achieve the steady insertion speed was measured by a Rotating Dynamometer (Type 9123B) (Kistler Instruments Ltd, Hampshire, UK) connected to a PC equipped with Dynoware program (Kistler Instruments Ltd, Hampshire, UK) for force signal acquisition and conditioning. The insertion performance was evaluated by contrasting the maximum axial insertion force in each test as well a post-insertion observation of the wear of treated and untreated pins using Toolmakers optical microscopy having maximum magnification of 400× and fitted with a camera for micrographs capturing.

### 2.5. Antibacterial Testing

Antibacterial performance was assessed of Unt, C2T620-80 and C3T660-20PdAg Ti-6Al-4V coupons (n = 3) using a methodology based on the Miles and Misra method [18]. Single colonies of gram-positive *Staphylococcus aureus* (*S. aureus*) (NCTC 6571) were serially diluted in trypton soya broth (TSB) down to 10^4^ from 10^9^ cells/mL. Each sample was inoculated with 20 µL of this solution and then incubated for 6 h before dilution in 10 mL of phosphate buffered saline (PBS) and vortexing to dislodge the surface adhered bacteria. Serial dilutions to dilute this solution 10^−7^ were then carried out in a 96 well plate, and 20 µl of each dilution factor from the 96 well plate was pipetted into a quadrant on a tryptone soya agar (TSA) plate of their respective dilution factor. These were then incubated at 37 °C overnight, after which each quadrant was observed for growth and colonies counted. The number of colony-forming units (CFU) per ml from the original aliquot was calculated from this.

## 3. Results

### 3.1. Optimising CCT with Catalytic Pre-Depostion Layer

#### 3.1.1. 80 h Treated Coupons at Temperatures of 580, 620 and 660 °C

**Figure 1:**
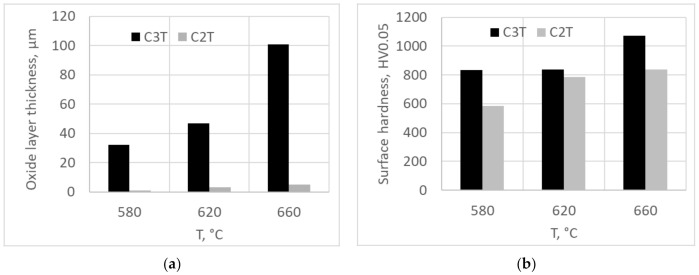
Oxide layer thickness (**a**) and surface hardness (**b**) of samples with and without a pre-deposition layer of Pd/Ag vs. ceramic conversion treatment of 80 h at different temperatures.

Coupons with and without a pre-deposition layer of Pd/Ag were treated for 80 h at temperatures of 580, 620 and 660 °C. Formed surface oxide layer thickness and corresponding surface hardness of the samples are charted in Figure 1a,b. It was found that the Pd/Ag pre-deposition layer played a catalytic role on the ceramic conversion treatment as the surface layer thickness significantly increased up to 32 times compared with the bare surface ceramic conversation treatments. Observations of the three different temperature treated coupons determined that the 660 °C treatment demonstrated a thick 100 µm hardened surface layer, possessing an over 1000 Vickers hardness with a good interfacial adhesion, which stands out as an ideal treatment temperature for further treatment development.

#### 3.1.2. Ceramic Conversion Treatment of 660 °C for a Series of Time Durations

**Figure 2:**
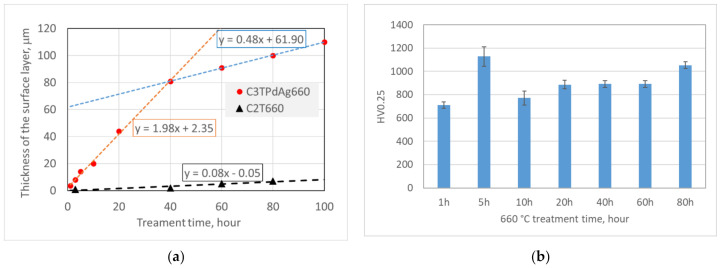
Surface layer (**a**) thickness of C3TPdAg660 and C2T660 samples and (**b**) hardness of C3TPdAg samples treated at 660 °C for different time periods.

Figure 2 charts the oxide layer thickness of C2T and C3T samples and surface hardness of the C3TPdAg coupons treated at 660 °C for different durations. It can be seen that the Pd/Ag pre-deposition layer resulted in a much thicker oxide layer than the coupons without pre-deposition. A significant catalytic effect of Pd/Ag on the formation of the oxide layer on Ti-6Al-4V was observed and a growth rate of about 2 µm/h was extrapolated from Figure 2a for C3T treated coupons when treated within 50 h. This was 25 times faster than that of the C2T treatment coupons within the same time period. When the treatment time increases to over 60 h, the oxide layer growth rate was reduced to 0.5 µm/h, but this is still 6 times higher than the growth rate of C2T samples. Surface hardness evaluation of C3T treated coupons revealed a generally increasing trend with increasing treatment duration to a maximum of 1053 HV0.25, except for the 5 h treated sample, which shows a relatively higher hardness value of 1128 HV0.25. It can be seen that a short treatment time of 5 h produced a 14.60 μm hardened surface oxide layer, which is one of the purposes of this study. Further investigation was conducted under the treatment condition of 660 °C 5 h with Ag or Pd pre-deposited, to investigate the catalytic effect of the individual elements.

#### 3.1.3. 660 °C 5 h Treatment of Ag, Pd and Ag + Pd Pre-Deposited Coupons

**Figure 3:**
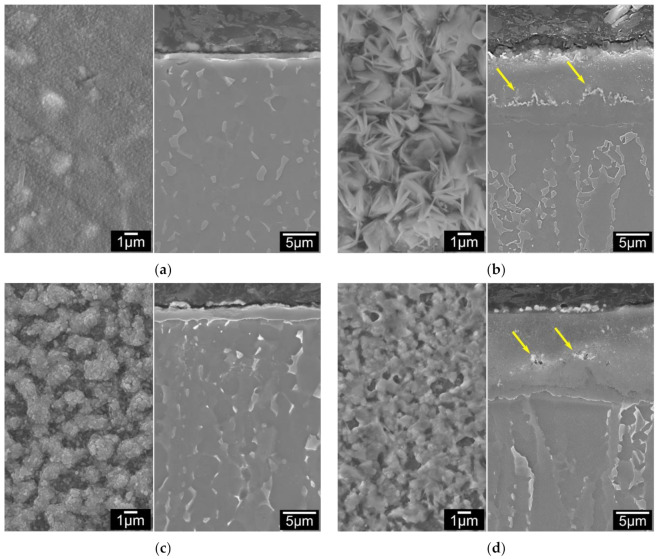
Surface morphology (left) and layer structure (right) of 660 °C, 5h treated Ti6Al4V samples: (**a**) no catalytic layer; (**b**) with Ag, (**c**) Pd and (**d**) PdAg catalytic pre-depositions.

Coupons with pre-deposited coatings of Ag, Pd and Ag/Pd were ceramic conversion treated at 660 °C for 5 h with the uncoated coupons for comparison. Figure 3a–d show the SEM images of these treated samples, where the left images are of surface morphology and right are of cross-section surface layer structures. It reveals that the 660 °C 5 h treated C2T sample was covered by a smooth and fine roundish shaped oxide (Figure 3a left). The surface morphology of the C3T samples presented different features depending on pre-coating element. For Ag pre-coated samples, the oxides presented in bunches of acicular grains (Figure 3b left). When Pd was pre-coated, the sample surface was covered by clusters of fine grains (200–300 nm) after the treatment (Figure 3c left). While for Ag and Pd co-deposited samples, oxides were shaped in micro-grains with a few thin needles (Figure 3d left). Corresponding cross-sectional microstructures of these four samples show a formed surface oxide layer in the thickness of 1.21, 11.40, 2.10 and 14.60 µm for C2T, C3TAg, C3TPd and C3TAgPd samples, respectively. For Ag and Ag + Pd pre-coated coupons, white contrasted particles (corresponding to the surface granular grains) are present on the outermost surface (Figure 3b,d) and some are imbedded within the oxide layer, as denoted by arrows. Surface roughness of the samples are varying from 0.082, 0.278, 0.090 to 0.349 µm for C2T, C3TAg, C3TPd and C3TPdAg samples, respectively.

Additionally, the surface hardness measured under 50 g show increased hardness of the samples with pre-coatings, especially for the co-deposited Pd/Ag samples.

Table 2 lists all observed data in terms of the surface layer thickness, hardness and roughness for these four samples. Summarising the observations, it is clear that the co-deposition of Pd/Ag not only catalyses oxide layer formation, but also produces an even harder layer than single element pre-deposition.

### 3.2. Microstructure Characterisation of Surface Oxide Layers

#### 3.2.1. Surface Morphology Evolution of C3T Pd/Ag Coupons

**Figure 4:**
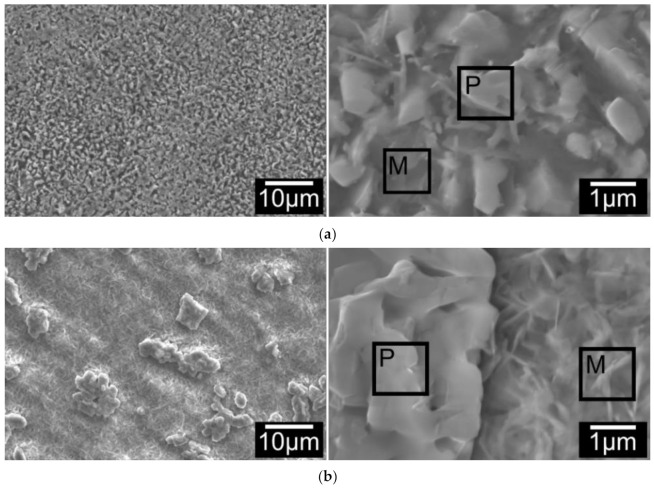

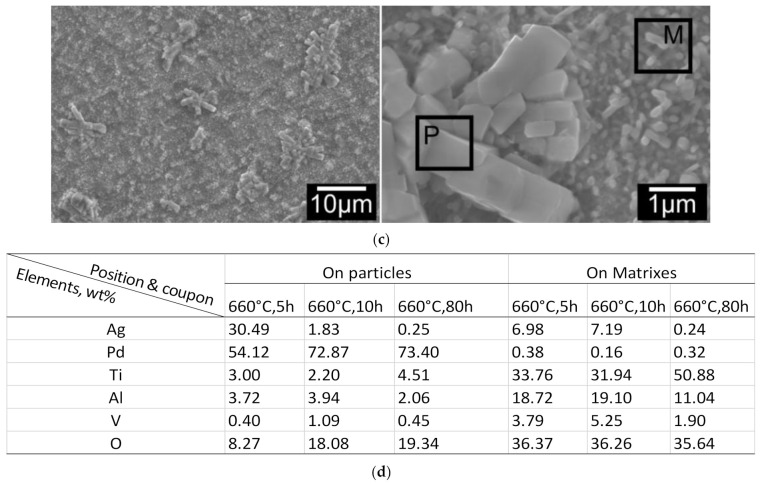
Surface morphology change of C3TAgPd coupons, treated at 660 °C for (**a**) 5 h; (**b**) 10 h and (**c**) 80 h. ‘P’ and ‘M’ denote particle and matrix, respectively; (**d**) elemental analysis on the spots of agglomerated particles and matrix grains for 5, 10 and 80 h treated coupons (see denoted P and M in a,b,c).

SEM observation on surfaces of all C3TPd/Ag coupons revealed that the surface morphology of the coupons present three typical features as shown in Figure 4a for treatment durations of 1–5 h, surface oxides shaped as micro-grains with a few thin needles; Figure 4b for treatment durations between 10 to 60 h, the surface is composed of agglomerated micro-grains and fine, disordered needles and the blocks of the agglomerated grains were reduced with increasing treatment duration; and Figure 4c over 60 h treatment durations, the agglomerated micro-grains transformed to faceted grains and the needles were changed to rods.

EDX mapping on the top surfaces of C3TPdAg coupons revealed elemental distribution along the surface features: all agglomerated/faceted micro-grains are composed of Ag, Pd and O, and acicular (or matrix) grains are detected as V, Ag, Al, O and Ti. The quantified EDX spot analysis on the agglomerated grains and the acicular/matrix grains for 5, 10, and 80 h treated coupons are listed in Figure 4d. By comparing the quantity change trends on the particles and matrixes, it is very interesting to see that it was the Pd which agglomerated during the treatments, evidenced by it presenting dominantly in the particles. While for Ag, it was initially present within the particles dominantly (30.49 wt% for 5 h), and then reduced to 1.83 and 0.25 wt% after 10- and 80 h treatments, respectively. Its distribution in the matrix ground was kept constant (≈7 wt%) within 10 h treatment and then reduced to 0.25 wt% for the 80 h treated coupon, indicating its diffusivity into the formed oxide layer. High Al content was observed on the matrix for all treated coupons, alluding to alumina forming at the surfaces.

#### 3.2.2. Phase Identification by XRD Patterns

**Figure 5:**
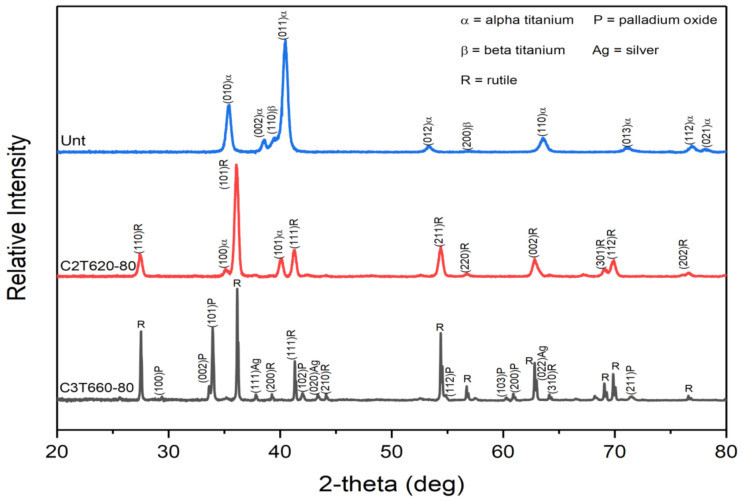
XRD patterns of as-received (Unt), C2T620-80 and C3T660-80PdAg samples.

XRD patterns of the typical C2T and C3TPdAg treated samples are shown in Figure 5 with untreated for comparison. As indexed, the untreated Ti-6Al-4V is composed of predominantly alpha-Ti phase and a few weak peaks of beta-Ti. After C2T treatment, rutile TiO_2_ phase was identified with weak peaks of alpha-Ti, which was contributed from the substrate as the formed oxide layer is only about 3 µm. For the C3T treated sample, in addition to the high intensity peaks of the rutile TiO_2_ phase, a new set of peaks was identified as a PdO phase (JCPDS 00-043-1024) and peaks of pure Ag phase were also detected. Some more peaks with low intensity could not be assuredly indexed to other oxides, such as Al_2_O_3_, AgO_2_ and V_2_O_5_ etc. phases.

#### 3.2.3. FIB/SEM and TEM Characterisation of the Surface Layer Structures

**Figure 6:**
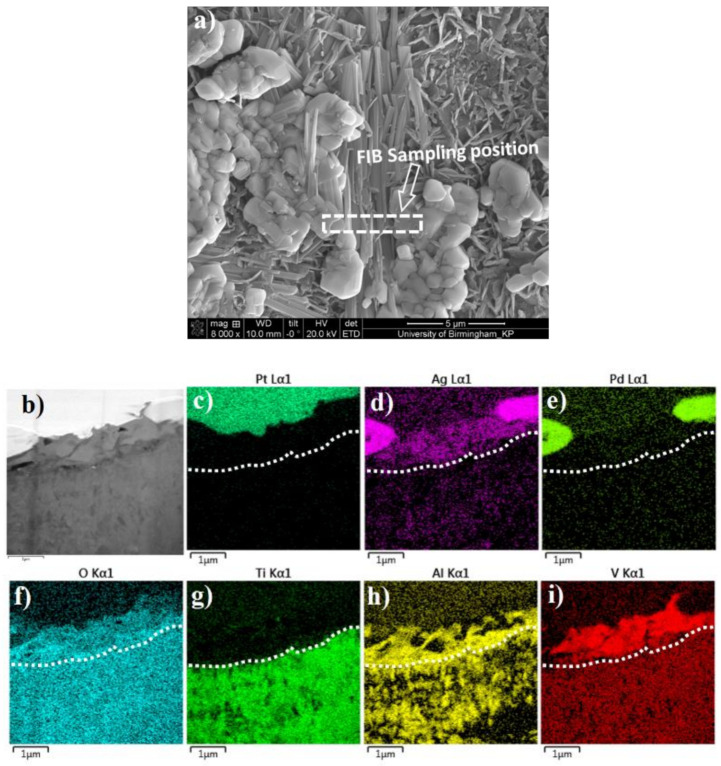
FIB sampling location (**a**); and HAADF image (**b**), of a TEM sample of C3T660-20AgPd coupon; (**c**–**i**) STEM-EDX element mapping for elements Pt, Ag, Pd, O, Ti, Al, V, respectively. Pt layer shown in (**c**) was deposited during the FIB sample preparation. A dash lines were added to indicate the boundary between the ‘catalytic’ topmost substances and the beneath TiO_2_ layer.

Further TEM characterisation was carried out on a C3T660-10PdAg coupon. A cross-section TEM sample was prepared by FIB, and the sampling position is across the agglomerated Pd (Ag) particle and the acicular matrix, as shown in Figure 6a. A STEM HAADF image and corresponding EDX elemental mappings of the TEM sample are shown in Figure 6b–i, which reveal cross-sectional surface oxide layer structures as shown by chemical composition distributions. An added dashed line on each element mapped image indicates the boundary between the topmost ‘catalytic‘ layer and the dominant TiO_2_ layer beneath. Combining the top morphology of the FIB sampling position (Figure 6a) with the cross-sectional view of the HDDF STEM image Figure 6b) and the EDX elemental mappings, it can be determined that: above the dashed line, the white contrasted grains at either side of the image (Figure 6b) are the agglomerated granular grains, and the grey middle area are acicular grains. EDX mappings show high intensities of Ag, Pd and O elements within the granular grains, while the needle shaped grains are indicating a composition of V, Ag, O, Al and Ti (trace). Interestingly, Al combined with O presented along the grain boundaries of the granular and acicular grains of the topmost layer. Beneath the topmost layer, compositional elements of alloy Ti-6Al-4V, Ti, Al, V, are all presented with O, Ag and Pd (low contrast).

**Figure 7:**
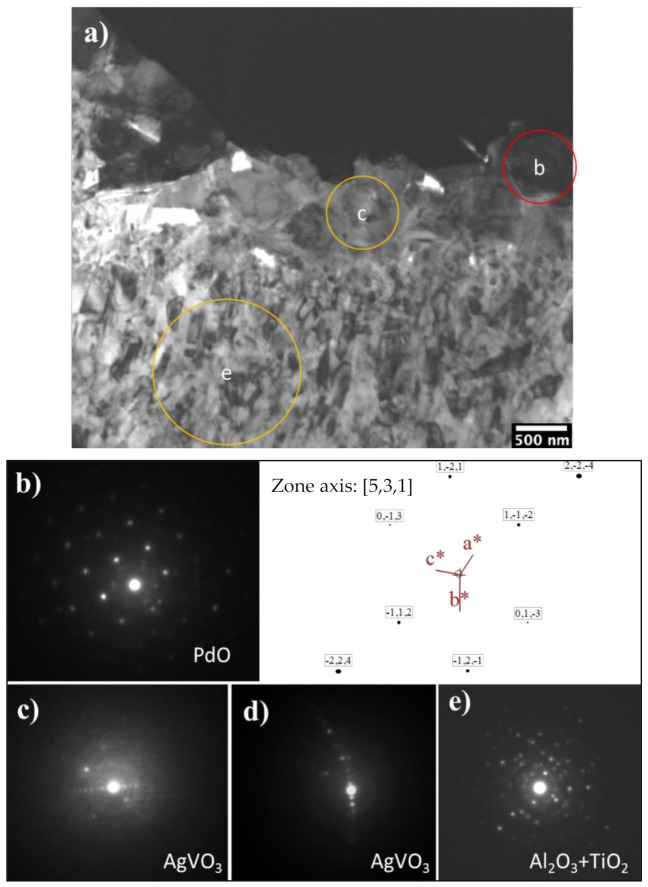
(**a**) BF-TEM of C3T660-10PdAg and corresponding SAD patterns. Region b corresponds to (**b**), region c corresponds to (**c**) and (**d**), and region e corresponds to (**e**). Areas of b, c, e are denoted in (**a**–**e**) indicate phases of PdO, AgVO_3_, TiO_2_ and Al_2_O_3_ as labelled.

Further TEM observation and SAD pattern analysis specified the phase composition of the oxide layer structures. As can be seen from Figure 7a, the surface oxide layer consists of a topmost layer with granular and acicular grains and a dominant columnar structured sublayer. It was observed that just beneath the topmost catalytic layer an approximately 500 nm thickness layer is present, with a nano-crystalline structure (50–100 nm diameter). Beneath this nano-grained layer, the columnar structure is evident and developed to the front of the substrate with column widths of approximately 100–200 nm and lengths of approximately 400–600 nm.

SAD patterns taken from the granular grains were identified as a PdO phase, evidenced by a B = [531] pattern shown in Figure 7a,b, which is consistent with the XRD peaks indexing of PdO in Figure 5. No Ag oxides could be identified from the diffraction patterns taken from the granular grains. As the EDX detected co-existing of Ag, 2 wt%, with Pd in the granular grains (see Figure 4d), a PdO-like oxide with some of the Pd being substituted by Ag, or a Pd(Ag)O phase, was considered for the granular grains. Another possibility of an Ag capping layer on top of the Pd particles cannot be ruled out. Prior studies show that PdAg surfaces can be highly dynamic at modest temperatures and heating to only 400 K in UHV promoted the formation of an Ag capping layer on top of the Pd islands, thereby lowering the surface free energy as the surface free energy of Pd is 1.6 times higher than that of Ag [19,20].

Indexing the SAD patterns of the acicular grains identified an AgVO_3_ (JCPDS 0029-1152) phase (Figure 7a,c,d). Faint diffraction rings were always superimposed in the Pd(Ag)O and AgVO_3_ patterns, which could be contributed to an amorphous or poorly crystalline structure of aluminium oxide based on the fact that Al was presented among the grain boundaries of these grains, as seen from the EDX mapping in Figure 6h, but it is difficult to identify the Al_2_O_3_ phase from the SAD patterns taken in these areas.

SAD patterns taken from the columnar-grained layer show some discrete rings, which denote the polycrystalline nature of the layer and one example is presented in Figure 7e). Indexing of the discrete rings identified a dominant rutile TiO_2_ phase with a few rings corresponding to Al_2_O_3_. Although EDX analysis shows Ag, Pd and V elements presenting (Figure 6d,e,i) within this layer, no other oxides could be identified from the SAD patterns taken from this layer, suggesting that these elements either present as substitute elements to Ti in the TiO_2_ phase or as pure elements. It was observed that the interface between the columnar oxide layer and the substrate are dense and without voids. The SAD patterns taken from the substrate adjacent to the oxide layer revealed an expanded HCP structure of α-Ti(O), or oxygen diffusion zone.

### 3.3. Mechanical and Tribological Properties of the Treated Coupons and Pins

From Section 3.1., it is found that co-depositing Pd/Ag on Ti-6Al-4V (Ti64) prior to ceramic conversion treatment can effectively catalyse surface oxide layer growth and the formed surface layer possesses a high hardness of 700–1100 HV 0.025 (Figure 2b). Table 2 compares surface micro-hardness of the typical C3T coupons with C2T coupons. It can be seen that the C2T and C3T treatments produced a hardened surface layer with the hardness value of 931 and 1128 HV 0.025, respectively. This is approximately 2.5 and 3.1 times the hardness values of the untreated Ti64 coupons (360 HV 0.025).

**Figure 8:**
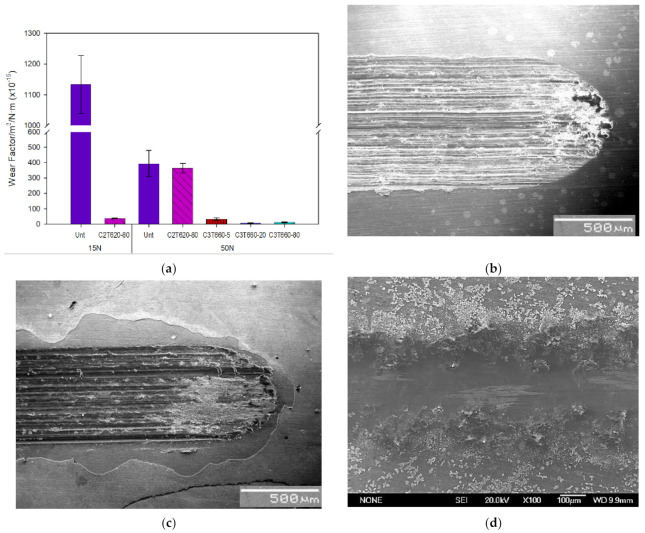
(**a**) Wear factors of Untreated, C2T and typical C3T coupons; SEM images of the wear tracks after 50 N wear tests for the coupons of (**b**) untreated; (**c**) C2T620-80 and (**d**) C3T660-5PdAg.

Reciprocating tests were carried out under two loads on selected C3T samples of 660 °C, 5, 20 and 80 h treated, compared with C2T treated and untreated (Unt) samples. When the wear tests were carried out under the normal load below 15 N, no measurable wear scars can be detected for both the C2T and C3T treated samples. The selected C3T coupons performed similar but excellent wear resistance under the two loads selected and the typical wear factor of the C3T coupons is compared with the C2T and untreated ones in Figure 8. It can be seen that under the normal load of 15N, there was no measurable wear for the C3T samples but the wear factor of C2T is ≈56 × 10^−15^ m^3^/N.m. It was only when the normal load increased to 50 N, a marginable wear scar could be seen and thus the wear factor can be measured/calculated for the C3T samples, which was 1/350 of the wear factor of C2T and Unt coupons. Compared with Unt Ti64, C2T demonstrated a 30 times reduction in wear factor when tested under the load of 15N. However, when further increasing the test load to 50 N, the improvement was marginal for C2T vs. Unt coupons. This is because the surface layer of C2T is very thin, and when it is tested with a heavy load, excessive plastic deformation in the substrate occurred, and the wear debris from the hardened surface acted as the third body. This resulted in the reduced tribological properties shown [21]. It should also be noted from Figure 8 that the wear factor is larger under the lower load of 15 N than under the higher load of 50 N for the Unt samples. This phenomenon is believed to be due to the fact that although there was a 3.3 times increase in load (15 to 50 N), there is not a 3.3 times increase in contact pressure. This is due to the untreated Ti64 undergoing more deformation at the higher load, thus increasing the contact area between the substrate and counterpart, therefore reducing the contact pressure. This is not taken into consideration in the wear factor calculation. Additionally, the higher load may have caused a sufficient temperature increase between the substrate and counterpart during wear, which induced the generation of a tribofilm consisting of oxidised Ti64 wear particles, thus increasing wear resistance of the Ti64 [22].

SEM observation of the wear tracks of the C3T660-5PdAg sample tested under 50 N revealed a polished smooth surface rather than a wear track as evidenced in Figure 8d. While for Unt and C2T samples, severe wear tracks were formed after the tests under 50 N. The Unt samples displayed severe adhesive wear characterised by the plastic deformation and adhesion junctions between the counterparts. In addition, the asperities of the counterpart, a WC ball, exerted a significant ploughing effect on the Unt sample, resulting in dense deep and wide grooves shown in Figure 8b, features indicating abrasive wear [23,24]. For C2T samples, the wear tracks featured surface oxide layer chip-off and abrasive wear groves, which was caused by the heavy load applied. During the wear tests, the counterpart wore through the oxide layer and the hard oxide debris acted as a third body, abrasively wearing into the underlying bulk material (Figure 8c).

**Figure 9:**
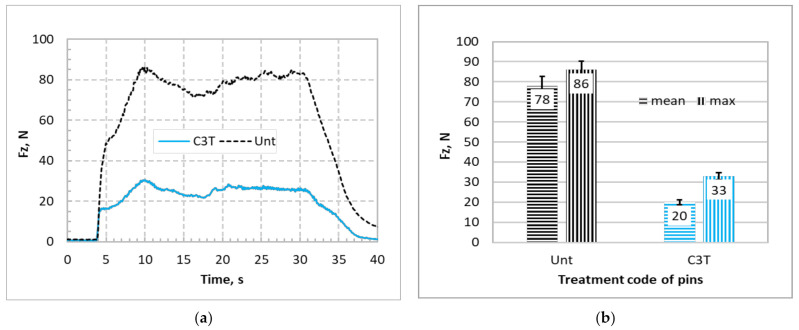
Comparisons of (**a**) recorded insertion forces against drilling time and (**b**) average and maximum insertion forces for untreated and C3T660-5PdAg treated self-drilling/self-tapping pins inserting into SAWBONES^®^ cortical bone plates.

**Figure 10:**
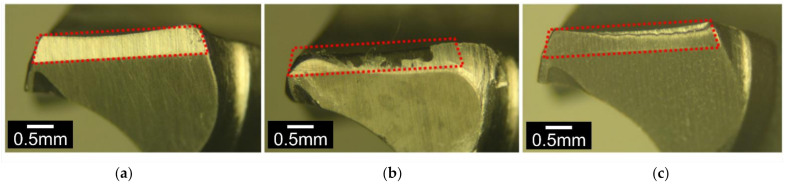
Optical images of cutting-edge areas of the pins: (**a**) as received new pin, (**b**) untreated, and (**c**) C3T660-5PdAg treated pins after insertion test with SAWBONES® cortical bone plates.

The recorded axial forces, Fz, needed to achieve the insertion distance of 25 mm through the bone plate against the insertion time are shown in Figure 9a for Unt and C3T660-5PdAg pins. It can be seen that for the Unt pins, the applied axial force increased linearly before it reached the maximum force value, then dropped to a nearly constant force, with some fluctuation as the pin went through the bone simulation block. Finally, it rapidly reduced before it flattened at a low value after the pin fully went through the bone plates. Compared with the Unt pins, a significant reduction in the insertion force was observed for C3T treated pins. As can be seen from Figure 9b that the mean/maximum insertion forces are 20/33 N for the treated, and 78/86 N for the untreated, a nearly four times reduction in mean insertional force of the C3T pins vs. the untreated pins. Post insertion test observation on the pin’s tip at the cutting edge revealed severe damage for the Unt pins compared with the C3T treated pins, as evidenced in Figure 10. Compared with the new pin in Figure 10a, the cutting edge of the Unt and C3T treated pins have worn off approximately 85% and 9% of the tip volume, respectively, indicating a great performance of the C3T treated pins over the Unt ones, which could be attributed to the larger difference in hardness between the C3T treated (1100 HV) and the untreated (370 HV) surfaces.

### 3.4. Anti-bacterial Efficacy

**Figure 11:**
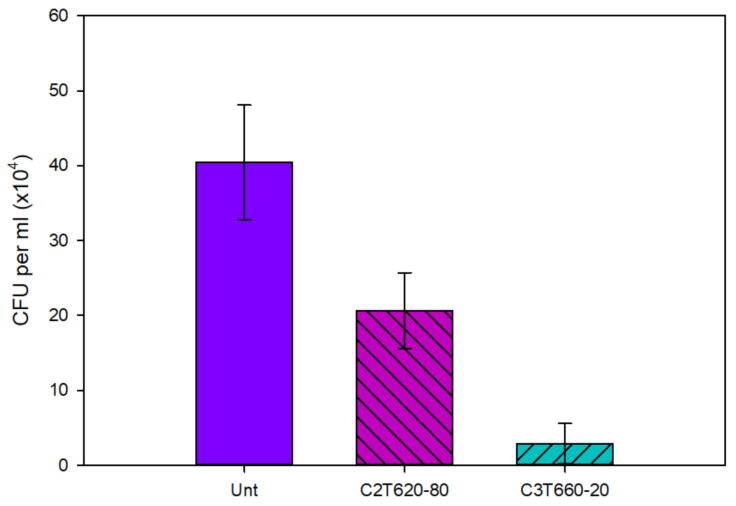
The antibacterial performance of untreated and treated Ti64. Where a smaller number of colony-forming units (CFU) is desirable. Note scale is multiplication.

The antibacterial performance of Unt and treated Ti64 coupons is shown in Figure 11. Post 6 h incubation, the average number of viable colony-forming units (CFU) per ml of *S. aureus* inoculant reduced significantly (P < 0.05) for both C2T and C3T coupons compared with Unt (40 × 10^4^) coupons. C3T demonstrated a further significant (P < 0.05) reduction vs. C2T, of 3 × 10^4^ vs. 20 × 10^4^, respectively. This clearly indicates that the ceramic conversion treatment can effectively improve the antibacterial efficacy of Ti64 alloy with approximately a 50% reduction in the number of CFUs. Furthermore, the catalytic elements introduce an even greater significant increase in antibacterial performance, with a reduction in CFUs of 93% compared with the Unt coupons.

## 4. Discussion

### 4.1. Catalytic Effect on the Growth of the Oxide Layer

It is clear from Figure 2a that coupons with a pre-deposition of a Pd/Ag layer significantly increased surface oxide layer growth rate by 25 times that of the C2T treatment coupons within the 50 h treatment durations. When the treatment duration increases to over 60 h, the oxide layer growth rate of C3T coupons reduced to 0.5 µm/h, but this is still 6 times higher than the growth rate of the C2T samples. SEM and TEM observation revealed the oxide layer surface morphology evolution and layer structure details. The catalytic effect of Pd/Ag on the fast growth of the oxide layer can be drawn from the following aspects:

#### 4.1.1. Surface Morphology Evolution of C3T Pd/Ag Coupons

For the Ti64 alloy, at low temperatures (or the initial oxidation period) in an oxidizing atmosphere, oxide scale formation follows these steps [1]: oxygen adsorption at the surface, oxide nucleation, lateral growth of the nuclei and formation of a compact oxide scale, which will then separate surface from the gaseous environment, thus retarding the oxidation processing. For pre-deposited Pd/Ag Ti64 samples, the surface deposition layer is very thin, which will not prevent titanium adsorption of oxygen. Instead, these nano-particles were blocking the lateral growth of the TiO_2_. This made the TiO_2_ grains form in the nano-scale, and in turn, those grain boundaries provided multiple tunnels for fast inward oxygen diffusion. Similar observations were reported by Prodromides et al. [25] on TiZrV getter films, where the presence of grain boundaries in the nanocrystalline structure promoted in depth diffusion of oxygen. This effect was continued until Pd particles were agglomerated to large particles and Ag was exhausted from the surface due to dispersion within the formed TiO_2_, as evidenced by the fact that the oxidation rate was reduced only after 60 h.

#### 4.1.2. Nano-Crystallisation of Columnar TiO_2_ Grains by Finely Distributed Ag Particles

STEM/EDX chemical composition mapping of the C3T660-20 PdAg sample (Figure 7) demonstrates movement of the pre-coated Pd/Ag catalytic elements for treatment durations longer than 10 h at 660 °C. It revealed that Pd agglomerated at the surface and formed PdO, while Ag particles were finely dispersed in the TiO_2_ layer, and the quantitative analysis of the Ag content in both particles and the matrix (TiO_2_) further confirmed this observation (Figure 4d). Based on the Ellingham-diagram [26], Ag_2_O can be formed at low temperatures when oxidised in air. However, it is unstable above 462 K and the formed Ag_2_O will reduce to stable Ag metal. Due to Ti’s strong affinity to O, when Ti diffuses outwards to meet oxygen and form TiO_2_, the stable Ag particles act as doping elements pinning TiO_2_ grains, thus preventing them growing to a large size. This resulted in the formation of nano-crystalline columnar TiO_2_. This formed near vertical (to the sample surface) columnar grain boundaries between the nano-crystalline grains, thus acting as oxygen diffusion channels. The dispersion of Ag particles in the TiO_2_ matrix are in good agreement with Adochite et al. [27] who reported on a silver doped TiO_2_ system obtained by magnetron sputtering after annealing between 400–600 °C, and such a phenomenon might have been induced by Ag diffusion along the TiO_2_ grain boundaries, as suggested by Armelao et al. [28].

On the other hand, the nano-crystaline columnar TiO_2_ grains prevented the formation of a lateral Al_2_O_3_ layer. According to Du et al. and Cimenoglu et al. [29,30] multi-layered oxides will form on the surface of Ti–6Al–V alloy with long duration oxidation, which is due to alternating growth of Al_2_O_3_ and TiO_2_ layers by outward diffusion of aluminium and inward diffusion of oxygen. At the early stages of oxidation, Al_2_O_3_ nucleates on the surface together with TiO_2_. At extended oxidation times, Al_2_O_3_ grows laterally and covers TiO_2_. In this case, the outer most section of the oxide layer (gas/oxide interface) is Al_2_O_3_, while the inner section of the oxide layer (oxide/substrate interface) is TiO_2_. Once the critical thickness is exceeded, cracks develop at the interface, where the conditions might be similar to the bulk atmosphere of the early stage of oxidation. Aluminium from the substrate would again diffuse outward and form the second Al_2_O_3_ layer on the second TiO_2_ layer in the crack. However, this lateral structure, and thus the cracks, were not observed within the C3T coupons even after 80 h treatment. The finely distributed Ag within columnar nano-crystalline TiO_2_ grains firstly prevented the lateral TiO_2_ oxide layer growth and secondly, the fast columnar growth of the TiO_2_ ejected the aluminium along the grain boundaries perpendicular to the surface, as evidence in Figure 6h.

Surface enrichment of vanadium, and thus the formed AgVO acicular phase, during ceramic conversion treatment may also contribute to the fast growth of the surface oxide layer. It is reported that the oxygen diffusion coefficient along grain boundary is 10^8^ times greater than that of bulk diffusion [31].

### 4.2. Improved Tribological and Antibacterial Properties of the C3T Surface Layer

The tribological properties of the C3T treated coupons and fixation pins are significantly improved compared with the untreated ones. With the WC ball counterpart, even under the normal load of 50N, or the maximum Hertzian contact pressure of ≈2000 MPa, for 1000 cycles, only a marginal wear track can be measured. This super wear resistance is contributed from the outermost layer structure, demonstrated in Figure 6 and Figure 7, which consists of agglomerates of palladium (with trace silver) oxide, vanadium oxide and amorphous-like fine grains of alumina presenting within the grain boundaries. This superficial layer with the underneath supporting nano-columnar TiO_2_ + Al_2_O_3_ layer provided high surface hardness of above 1000 HV 0.05 and high load bearing capacity, which outperforms the poor wear resistance of the C2T treated sample under the heavy load of 50 N as evidence in Figure 8c, where spallation of the thin surface oxide layer and deep groves of adhesive and abrasive wear can be seen. The excellent wear performance of the treated fixation pins further proved that the C3T treatment is well suited for complexly shaped components and the loading forces, as post-drilling testing SEM observation revealed no surface damage along the body of the pins, and very minor wear at the tip of the pins (Figure 10c).

Within the biomedical field, implant-associated infections are a major concern. Therefore, the ability of a material to demonstrate antibacterial behaviour is important for reducing implant failure rates in order to reduce both patient and NHS stress. The antibacterial properties of the C2T compared with untreated Ti64 (Figure 11) demonstrated a significant reduction, of approximately 50%, in the amount of *S. aureus* CFU per ml. This is consistent with the literature [12,32]. This is thought to be due to titanium oxide inherently being able to produce reactive oxygen species (ROS), capable of disrupting cell membranes and walls.

A further reduction in the number of CFU per ml was found with C3T. Where compared with Unt, there was a 93% reduction, and when compared with C2T, an 85% reduction. This is since it has silver present on the surface, which is well known for its antimicrobial activity. This is thought to be from hindering metabolism and DNA replication of the bacterial cells, ultimately resulting in cell death. [13,14,32,33]

These findings provide evidence that the reported treatments can produce a durable surface, capable of reducing the number of CFU/mL of *S. aureus* without the need for additional antimicrobial coatings/material, such as biomimetic anchored polymer brushes [34] or antimicrobial Ag/polyacrylonitrile nanofibers [35], which although they offer antibacterial activity, would not offer a durable antimicrobial solution for orthopaedic implants. To this end, further testing with different bacterial strains is merited.

## 5. Conclusions

A novel catalytic ceramic conversion treatment on Ti-6Al-4V was designed and optimised to produce a surface layer with excellent tribological and antibacterial properties for biomedical applications. The C3T was carried out by applying thin catalytic films of silver and palladium onto the Ti64 surface and then oxidising at 660 °C for a variety of hours. XRD, SEM, TEM and EDX analysis revealed a surface layer structure consisting of two sublayers: (1) a superficial layer with agglomerates of palladium (with trace silver) oxide, vanadium oxide, and amorphous-like fine grains of alumina presenting within the grain boundaries; (2) a dominant nano-columnar TiO_2_/Al_2_O_3_ layer with traces of silver and palladium particles. The PdAg catalytic effect on the growth of the surface oxide layer has been shown to significantly increase the oxide thickness. When treated for less than or more than 60 h it is 25 or 6 times faster, respectively, than that of the C2T treatments within the same treatment duration.

Surface hardness of C3T treated coupons increased from C2T of ≈900 to C3T of ≈1100 HV0.025. Excellent tribological performances were also observed for C3T treated coupons compared with both untreated and C2T, with a reduction of wear factors from ≈390 to ≈5 m^3^/N·m. The drilling tests of the fixation pins proved the resilience of the C3T treatment for complex shaped components and forces. It is observed that the mean insertion force of the C3T pins is nearly four times lower than that of the untreated pins. Additionally, a significant reduction in the number of colony-forming units per ml of *Staphylococcus aureus* on the C3T surfaces was observed.

## Figures and Tables

**Table 1:** Treatment conditions for the coded samples.

Code	Catalytic Layer	Treatment, T-t (°C-h)
Unt	-	-
C2T580-80	No	580-80
C2T620-80	No	620-80
C2T660-80	No	660-80
C3T580-80PdAg	Pd + Ag	580-80
C3T620-80PdAg	Pd + Ag	620-80
C3T660-80PdAg	Pd + Ag	660-80
C3T660-hPdAg	Pd + Ag	660-1,5,10,20,40,60,80
C2T	No	660-5
C3TAg	Ag	660-5
C3TPd	Pd	660-5

**Table 2:** Surface layer thickness, hardness and roughness for 660 °C-5 h treated samples with and without pre-coating elements (for sample code details see Table 1).

660-5h	Thickness, µm	HV0.05	HV0.025	Ra, µm
C2T	1.21	612 +/− 34	931 +/− 20	0.082 +/− 0.005
C3TAg	11.4	720 +/− 52	1067 +/− 72	0.278 +/− 0.037
C3TPd	2.10	686 +/− 96	1099 +/− 68	0.090 +/− 0.011
C3TPdAg	14.6	853 +/− 51	1128 +/− 82	0.349 +/− 0.025

## Data Availability

Data available in a publicly accessible repository.
